# Genome-wide profiling of nardilysin target genes reveals its role in epigenetic regulation and cell cycle progression

**DOI:** 10.1038/s41598-017-14942-4

**Published:** 2017-11-01

**Authors:** Yusuke Morita, Mikiko Ohno, Kiyoto Nishi, Yoshinori Hiraoka, Sayaka Saijo, Shintaro Matsuda, Toru Kita, Takeshi Kimura, Eiichiro Nishi

**Affiliations:** 1 Department of Cardiovascular Medicine, Kyoto University Graduate School of Medicine, 54 Shogoin-Kawahara-cho, Sakyo-ku, Kyoto, 606-8507 Japan; 2 Department of Pharmacology, Shiga University of Medical Science, Seta Tsukinowa-cho, Otsu, 520-2192 Japan; 3 Division of Clinical Pharmacy, Faculty of Pharmaceutical Sciences, Kobe Gakuin University, Chuo-ku, Kobe, 650-8586 Japan; 4 Kobe Home Medical and Nursing Care Promotion Foundation, 14-1 Naka Ichiriyama, Kami Aza, Shimotani, Yamada-cho, Kita-ku, Kobe, 651-1102 Japan

## Abstract

Post-translational histone modifications, such as acetylation and methylation, are prerequisites for transcriptional regulation. The metalloendopeptidase nardilysin (Nrdc) is a H3K4me2-binding protein that controls thermoregulation and β-cell functions through its transcriptional coregulator function. We herein combined high-throughput ChIP-seq and RNA-seq to achieve the first genome-wide identification of Nrdc target genes. A ChIP-seq analysis of immortalized mouse embryo fibroblasts (iMEF) identified 4053 Nrdc-binding sites, most of which were located in proximal promoter sites (2587 Nrdc-binding genes). Global H3K4me2 levels at Nrdc-binding promoters slightly increased, while H3K9ac levels decreased in the absence of Nrdc. Among Nrdc-binding genes, a comparative RNA-seq analysis identified 448 candidates for Nrdc target genes, among which cell cycle-related genes were significantly enriched. We confirmed decreased mRNA and H3K9ac levels at the promoters of individual genes in Nrdc-deficient iMEF, which were restored by the ectopic introduction of Nrdc. Reduced mRNA levels, but not H3K9ac levels were fully restored by the reintroduction of the peptidase-dead mutant of Nrdc. Furthermore, Nrdc promoted cell cycle progression at multiple stages, which enhanced cell proliferation *in vivo*. Collectively, our integrative studies emphasize the importance of Nrdc for maintaining a proper epigenetic status and cell growth.

## Introduction

Regulated gene transcription is crucial for cellular differentiation, proliferation, and homeostasis. Post-translational histone modifications, such as acetylation, methylation, and phosphorylation, may mark the functional regions of chromatin and are prerequisites for transcriptional regulation^1,2^. For example, the methylation of histone H3K4, particularly mono-, di-, and tri-methylation (H3K4me1, H3K4me2, and H3K4me3, respectively), correlates with active gene transcription, whereas H3K9 tri-methylation correlates with gene silencing and heterochromatin formation^3–5^. Acetylated H3K9, 14, 27 accumulate around transcription start sites (TSS) and/or enhancers of transcriptionally active genes and are generally associated with transcriptional activation by recruiting effector proteins that harbor acetyl-binding domains^6,7^. An open chromatin state is established by interactions among multiple chromatin-modifying enzymes, epigenetic regulators, and transcription factors.

Nardilysin (N-arginine dibasic convertase, Nrdc) is a metallopeptidase of the M16 family that cleaves dibasic sites^8^. We identified Nrdc as a receptor for heparin-binding EGF-like growth factor (HB-EGF)^9^. Our subsequent studies revealed that Nrdc enhances the ectodomain shedding of not only HB-EGF, but also other membrane proteins, such as tumor necrosis factor alpha (TNF-α) and amyloid precursor protein (APP), at the cell surface^10–14^. Besides its extracellular function, we recently demonstrated that Nrdc in the nucleus functions as a transcriptional coregulator^15–17^. Nrdc preferentially binds to H3K4me2 and, to a lesser extent, H3K4me3 among methylated histone H3 tails *in vitro* and *in vivo* ^15^. Nrdc either positively or negatively regulates gene transcription presumably via protein-protein interactions. Nrdc interaction with NCoR/SMRT/HDAC3 corepressor on proximal promoter on target genes in MEF^15^ and interaction with PGC-1α coactivator on Ucp1 enhancer in brown adipocytes repress transcription^16^, while Nrdc interaction with islet-1 on Mafa enhancer in pancreatic β-cell activates transcription^17^. Nrdc-deficient (Nrdc−/−) mice consistently exhibited a wide range of phenotypes including growth retardation, hypomyelination, hypothermia, and glucose intolerance^13,16,17^. Nrdc has also been reported to be strongly expressed in cancer tissues or cells in breast, gastric, and esophageal cancers^18–20^. These findings indicate the critical roles of Nrdc in homeostasis and suggest that Nrdc regulates a diverse set of genes depending on the cellular context^13^. Nevertheless, an analysis of Nrdc-binding sites in genomes and Nrdc target genes at the genome-wide level has not yet been conducted.

In the present study, we combined high-throughput chromatin immunoprecipitation-sequencing (ChIP-seq) and RNA-sequencing (RNA-seq) analyses to achieve the first genome-wide identification of Nrdc target genes. We used immortalized mouse embryo fibroblasts (iMEF) to investigate how Nrdc behaves in differentiated normal cells, given its status is readily usable for subsequent verifications. Epigenetic changes in H3K9 acetylation (H3K9ac) and H3K4me2 in the absence of Nrdc were also characterized. We found that global H3K9ac levels at Nrdc-binding promoters were increased and that a set of genes integral to cell cycle maintenance was directly activated by Nrdc. Collectively, these results suggest that the Nrdc-mediated epigenetic regulation of cell cycle-associated genes is a fundamental component of the biological functions of Nrdc.

## Results

### Nrdc-binding sites mainly localize in active promoter regions

**Figure 1:**
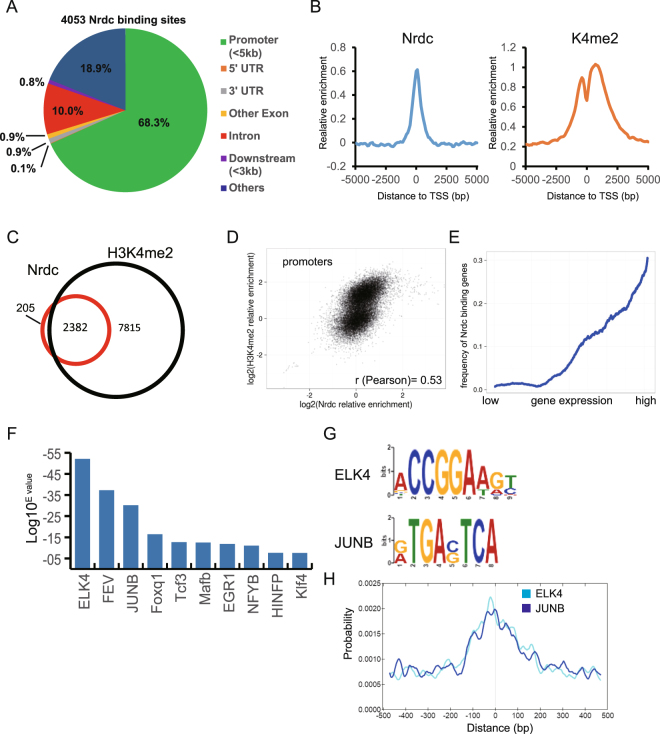
Nrdc primarily binds a subset of transcriptionally active promoters. (**A**) Genomic location of Nrdc ChIP-seq peaks in Nrdc+/+ iMEF with respect to the annotated genes. (**B**) Distribution of Nrdc and H3K4me2 around the TSS of 22656 of Ensembl protein-coding genes within 5 kb. (**C**) Venn diagram showing overlap between Nrdc-binding promoters (red) and H3K4me2-enriched promoters in Nrdc+/+ iMEF. The numbers of promoters in each section are shown. (**D**) Relationship between Nrdc and H3K4me2 relative enrichment in the promoters of Ensembl genes. (**E**) Ensembl genes were binned into every ten quantiles based on gene expression levels. The y-axis shows the percentage of Nrdc-binding genes in each quantile. (**F**) Identification of TF-binding sites enriched within Nrdc ChIP-seq peaks using an MEME-ChIP analysis. ELK4; ETS transcription factor, FEV; FEV (ETS oncogene family), JUNB; jun B proto-oncogene, Foxq1; forkhead box Q1, Tcf3; Transcription factor 3, Mafb; v-maf musculoaponeurotic fibrosarcoma oncogene family protein B, EGR1; early growth response 1, NFYB; nuclear transcription factor-Y beta, HINFP; histone H4 transcription factor, Klf4; Kruppel-like factor 4. (**G**,**H**) The sequences and distributions of ELK4 and JUNB motifs showing central enrichment within Nrdc-binding sites.

In order to obtain novel insights into the transcriptional role of Nrdc at the genome-wide level, we performed a ChIP-seq analysis using iMEF derived from wild-type mice (Nrdc+/+ iMEF). We generated an anti-mouse Nrdc monoclonal antibody by immunizing Nrdc-deficient mice with a recombinant protein corresponding to the C-terminal region of mouse Nrdc. We used the C-terminal fragment because all previous immunizations of wild-type animals (mouse, rat, and rabbit) with full-length Nrdc resulted in the production of antibodies recognizing the N-terminal fragment containing the highly acidic domain^21^, which did not work well in ChIP. We successfully obtained three clones of the anti-C terminus antibody, one of which showed high specificity and efficiency for cross-linked samples in immunoblotting and ChIP (Fig. S1A,B). The specificity of the antibody was also validated by ChIP-PCR targeting several genes randomly selected from H3K4me2-enriched genome regions in Nrdc+/+ and Nrdc−/− iMEF (Fig. S1C,D). Our ChIP-seq analysis of wild-type iMEF identified 4053 Nrdc-binding sites. A distribution analysis of Nrdc-binding sites in genomic regions demonstrated that most Nrdc-binding sites (68.3%) were located in proximal promoter sites, which are defined as locations within 5 kb of TSS (Fig. 1A and Table S2). By associating Nrdc-binding sites in proximal promoter sites with specific 22656 Ensembl protein-coding genes, we identified 2587 genes as “Nrdc-binding genes”. In order to characterize the functional categories enriched in these genes, a gene ontology analysis was performed using the DAVID Categories of fundamental biological processes^22^, such as translation, RNA processing, chromatin organization, and cell cycle, which were highly overrepresented in iMEF (Table S3).

We previously reported that Nrdc directly and specifically binds to H3K4me2^15^. Furthermore, we identified several target genes repressed by Nrdc, in which Nrdc is generally associated with a reduced level of H3 acetylation^15^. In order to characterize the relationship between Nrdc-binding sites and histone modifications, we performed ChIP-seq of H3K4me2 and H3K9ac in wild-type iMEF. We also investigated this relationship using previously published iMEF ChIP-seq datasets^23^. As a result, we found that 92.1% of Nrdc-binding genes contained H3K4me2 marks (Fig. 1C and Table S4). In addition, Pearson’s correlation analysis revealed that relative Nrdc enrichment around TSS correlated with H3K4me2 enrichment (Fig. 1D). On the other hand, the pattern of enrichment around TSS appeared to be slightly different between H3K4me2 and Nrdc. While the relative enrichment of the H3K4me2 signal decreased over TSS, as previously described^24^, the Nrdc signal peaked at the center of TSS (Fig. 1B), suggesting that Nrdc preferentially binds to the core promoter region. Other than H3K4me2, we found correlations between Nrdc-binding sites and active histone marks including H3K9ac and H3K4me3 (Fig. S2 and Table S5). In contrast, the negative histone mark of H3K27me3 did not show any correlation with Nrdc around the promoters. In order to further confirm whether Nrdc-enriched promoters were in the open state, we performed RNA-seq for wild-type iMEF and analyzed the relationship between Nrdc ChIP-seq signals and gene expression levels. As expected, Nrdc was more likely to bind to the promoter of genes with medium to high expression levels (Fig. 1E). These results indicate that Nrdc primarily localizes to active promoter regions.

We then performed a *de novo* motif analysis of MEME-ChIP to gain insights into the mechanistic and functional links between Nrdc and transcriptional factors (TF)^25^. Many binding motifs of transcriptional factors (ELK4, FEV, JUNB, Foxq1, Tcf3, Mafb, EGR1, NFYB, HINFP, and Klf4) were found in Nrdc-binding sites (Fig. 1F). Among them, a CentriMo analysis^26^ identified two binding motifs of ELK4 and JUNB (Fig. 1G,H), which were centrally enriched in Nrdc-binding sites. These results suggested the possibility that Nrdc interacts with these TFs. ELK4, a member of the Ets family, and JUNB, a binding partner of the AP-1 family, have been reported to be involved in a wide range of biological processes including cell growth, differentiation, and apoptosis^27,28^. Gene annotation by BioGPS^29^ indicated that Nrdc is ubiquitously expressed in mammalian tissues, while ELK4 and JUNB are highly enriched in mast cells and macrophages, respectively, suggesting the overlapped expression of these TFs and Nrdc (Fig. S3A–C).

### Combined ChIP-seq and RNA-seq analysis to identify direct Nrdc targets

In order to clarify how the deletion of Nrdc affects gene transcription and histone modifications, we performed a genome wide analysis of iMEF isolated from Nrdc-deficient mice (Nrdc−/− iMEF). As an optimal control, we prepared Nrdc−/− iMEF in which either wild-type or enzymatically inactive Nrdc^16,30^ was reintroduced by lentiviral-mediated gene rescue (Nrdc−/−^WT^ iMEF, Nrdc−/−^EA^ iMEF). Immunoblotting and immunocytochemistry showed that Nrdc expression levels in Nrdc−/−^WT^ iMEF and Nrdc−/−^EA^ iMEF were similar to those in wild-type iMEF (Nrdc+/+ iMEF) (Fig. S4A,B). As previously described, immunocytochemistry showed that endogenous and exogenous Nrdc are expressed in the nucleus and cytosol^15,16^ (Fig. S4B).

**Figure 2:**
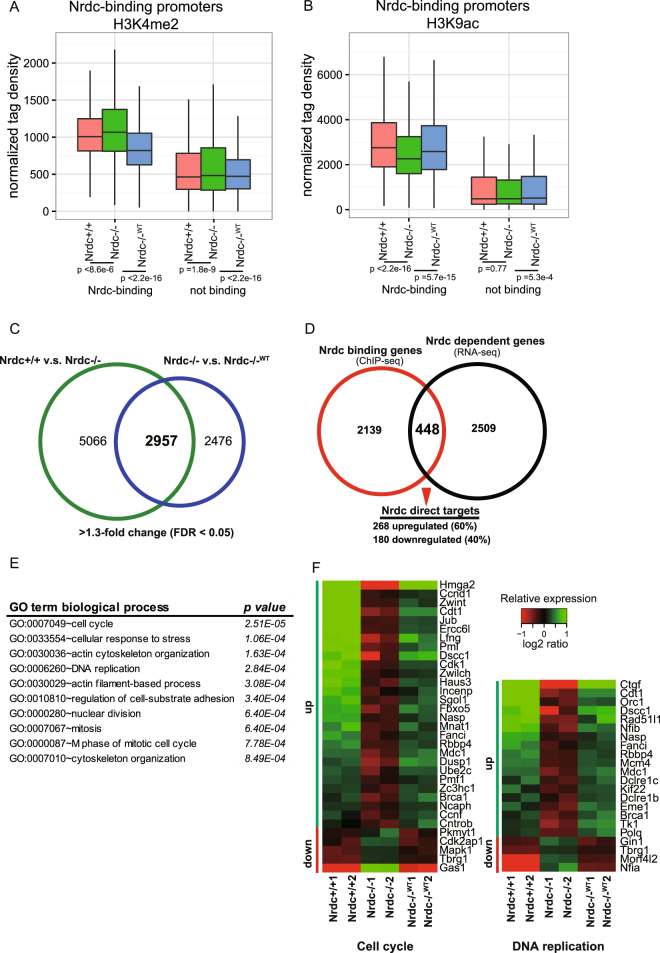
Nrdc affects the acetylation and methylation of histone H3 and gene transcription. (**A**,**B**) Average signals of H3K4me2 (**A**) and H3K9ac (**B**) from −5 kb to+5 kb surrounding the 2587 Nrdc-binding and other non-binding promoters in Nrdc+/+, Nrdc−/−, and Nrdc−/−^WT^ iMEF, which were normalized to total reads and read lengths. (**C**) Venn diagram showing the overlap of genes that were up- or down-regulated 1.3-fold or more in the same direction in the Nrdc+/+ v.s. Nrdc−/− group and Nrdc−/−^WT^ v.s. Nrdc−/− group. (**D**) Overlap between all Nrdc-binding genes and genes differentially expressed in an Nrdc-dependent manner, which identified a set of 448 Nrdc direct targets, with 268 being up-regulated and 180 being down-regulated. (**E**) Biological process GO terms enriched in the list of Nrdc direct target genes. P values are calculated by DAVID using a modified Fisher exact test^22^. (**F**) Heatmap showing the expression of genes within the categories of the cell cycle and DNA replication in Nrdc+/+, Nrdc−/−, and Nrdc−/−^WT^ iMEF. The color key indicates log2 transformed and mean-centered rpkm of genes. The color bars indicate the direction of either significantly up-regulated (green) or down-regulated (red) change in corresponding genes (FDR < 0.05 and fold change > 1.3).

A ChIP-seq analysis of H3K4me2 in Nrdc+/+, Nrdc−/−, and Nrdc−/−^WT^ iMEF revealed that global H3K4me2 levels at promoter regions were significantly decreased in the presence of Nrdc (Fig. 2A). The difference between Nrdc-deficient (Nrdc−/−) and Nrdc-expressing cells (Nrdc+/+, Nrdc−/−^WT^) was larger at Nrdc-binding promoters than at other promoters. In contrast, genome-wide H3K9ac levels at Nrdc-binding promoters were significantly higher in Nrdc-expressing cells than in Nrdc-deficient cells. No significant difference was observed in H3K9ac levels at non-Nrdc-binding promoters between Nrdc-expressing and Nrdc-deficient cells (Fig. 2B). These results indicate that Nrdc is involved in histone modifications.

In an attempt to identify which Nrdc-binding genes are transcriptionally regulated by Nrdc, we performed RNA-seq of Nrdc+/+, Nrdc−/−, and Nrdc−/−^WT^ iMEF. Using R library edgeR with a false discovery rate (FDR) of 0.05 and fold change of 1.3 or higher, we found 8023 significant differentially expressed genes (DEG) upon the Ensembl gene list between Nrdc+/+ and Nrdc−/− iMEF. There were also 5433 DEG between Nrdc−/−^WT^ and Nrdc−/− iMEF. Since genes showing the same direction of expression change in Nrdc+/+ and Nrdc−/−^WT^ iMEF are dependent on Nrdc, we focused on the 2957 overlapping DEG, which were similarly regulated by endogenous or ectopic Nrdc (Fig. 2C and Table S6). Among them, 1418 genes were upregulated, while 1539 genes were downregulated in the presence of Nrdc (Fig. S5A).

Among 2957 DEG, 448 genes (15%) were Nrdc-binding genes, which may be direct transcriptional targets of Nrdc (Fig. 2D and Table S7). Eighty-five percent of DEG were not identified as Nrdc direct targets, which implies that most DEG are regulated either by secondary effects or other non-transcriptional functions of Nrdc. Among 448 Nrdc direct target genes, 268 (60%) were up-regulated and 180 (40%) were down-regulated by the presence of Nrdc. We subjected the list of 448 Nrdc direct targets to a DAVID gene ontology (GO) terms analysis in order to investigate the molecular and cellular processes downstream of Nrdc direct targets^22^. As a result, a set of Nrdc target genes were enriched for several GO terms such as the cell cycle and DNA replication (Fig. 2E and Table S8). Most genes related to the cell cycle (28 out of 33) and DNA replication genes (18 out of 22) were up-regulated (Fig. 2F). Expectedly, separate DAVID analysis on up-regulated and down-regulated targets revealed that cell cycle and DNA replication genes were enriched in up-regulated direct targets, but not in down-regulated targets (Tables S7 and S9). On the other hands, genes related, for example, to steroid biosynthetic process were enriched only in down-regulated targets (Table S10). Moreover, comparative analysis of H3K4me2 and H3K9ac signals among the promoters of i) up-regulated (268), ii) down-regulated direct targets (180) and iii) non-direct targets (2139) demonstrated the higher enrichment of H3K9ac in up-regulated targets and lower enrichment of H3K4me2 in down-regulated targets in the presence of Nrdc (Fig. S5B,C). These data suggested that Nrdc activates or represses gene transcription via different epigenetic regulation.

### Nrdc transcriptionally regulates cell cycle-associated genes via the modulation of histone acetylation

**Figure 3:**
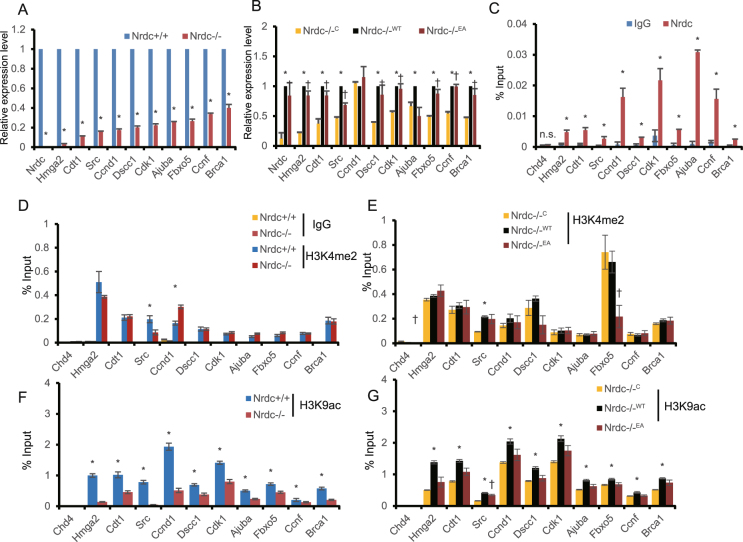
Nrdc transcriptionally regulates cell cycle-associated genes via the modulation of histone acetylation. (**A**, **B**) RT-PCR of the indicated cell cycle-associated genes in Nrdc+/+ and Nrdc−/− iMEF (**A**), and in Nrdc−/−^C^, Nrdc−/−^WT^, and Nrdc−/−^EA^ iMEF (**B**) (n = 3 per group). (**C**) ChIP-PCR using anti-Nrdc or control IgG shows Nrdc enrichment at the promoters of the indicated Nrdc direct targets in Nrdc+/+ iMEF (n = 3 per group). (**D**,**E**) ChIP-PCR analysis using anti-H3K4me2 and control IgG in Nrdc+/+ and Nrdc−/− iMEF (**D**), and in Nrdc−/−^C^, Nrdc−/−^WT^, and Nrdc−/−^EA^ iMEF (**E**) for the promoters of the indicated Nrdc target genes (n = 3 per group). (**F**,**G**) ChIP-PCR using anti-H3K9ac in Nrdc+/+ and Nrdc−/− iMEF (**F**), and in Nrdc−/−^C^, Nrdc−/−^WT^, and Nrdc−/−^EA^ iMEF (**G**) for the promoters of the indicated Nrdc target genes (n = 3 per group). All error bars indicate the standard error (S.E). *Indicates p < 0.05 between either Nrdc+/+ v.s. Nrdc−/− or Nrdc−/−^C^ v.s. Nrdc−/−^WT^. ^†^Indicates P < 0.05 Nrdc−/−^C^ v.s. Nrdc−/−^EA^.

In order to confirm the results of RNA-seq, we evaluated the mRNA levels of 10 selected Nrdc direct target genes related to the cell cycle (Cdk1, Fbxo5, Brca1, Hmga2, Cdt1, Src, Ajuba, Dscc1, Ccnf, and Ccnd1) in Nrdc+/+ and Nrdc−/− iMEF by real-time reverse transcription PCR (RT-PCR) (Fig. 3A). In all genes, we confirmed significantly stronger expression in Nrdc+/+ iMEF than in Nrdc−/− iMEF (Fig. 3A). Furthermore, the ectopic reintroduction of Nrdc in Nrdc−/− iMEF (Nrdc−/−^WT^ iMEF) also augmented cell cycle-related gene expression, except for Ccnd1, suggesting that Nrdc directly contributes to the transcriptional activation of these genes (Fig. 3B). In order to examine the contribution of the peptidase activity of Nrdc to transcriptional regulation, we also analyzed mRNA levels in Nrdc−/−^EA^ iMEF expressing the enzymatically inactive mutant of Nrdc (Fig. 3B). We found that Ccnd1 was not significantly up-regulated in Nrdc−/−^WT^ and Nrdc−/−^EA^ iMEF, suggesting that Ccnd1 expression is not strictly Nrdc-dependent. Ajuba was up-regulated in Nrdc−/−^WT^ iMEF, but not in Nrdc−/−^EA^ iMEF, indicating that its gene induction is dependent on the enzymatic activity of Nrdc.

Our ChIP-seq results demonstrated the association of Nrdc mostly around the TSS of cell cycle-related genes (Fig. S6). We also observed the presence of H3K4me2 and H3K9ac near TSS (Fig. S6). In order to confirm these ChIP-seq results, we performed site-specific ChIP-PCR using primers targeting Nrdc-binding sites in Nrdc+/+ iMEF. The anti-Nrdc antibody showed significantly stronger signals than control IgG. Negative control primers targeting the non-Nrdc-enriched region (Chd4) also gave no signals, confirming the specific recruitment of Nrdc to the identified Nrdc-binding sites (Fig. 3C).

Our ChIP-seq results demonstrated that Nrdc reduces global H3K4me2 levels and increases H3K9 acetylation levels at Nrdc-binding promoters (Fig. 2B). One possible hypothesis is that Nrdc activates gene transcription by regulating histone modifications at Nrdc-binding promoters. In order to test this hypothesis in individual target genes associated with the cell cycle, we performed site-specific ChIP-PCR using the anti-H3K4me2 or anti-H3K9ac antibody. Despite the increased expression of these Nrdc direct targets, H3K4me2 levels at the promoters were not changed, except for Src (Fig. 3D,E). This is consistent with previous findings showing lack of a causal relationship between histone methylation levels and gene expression^31^. On the other hand, H3K9ac levels at the promoters were significantly higher in Nrdc+/+ iMEF than in Nrdc−/− iMEF, which is consistent with higher gene expression levels in Nrdc+/+ iMEF. Moreover, the ectopic expression of Nrdc in Nrdc−/− iMEF restored decreased H3K9ac levels (Fig. 3F,G). Reduced H3K9ac levels were not fully restored by the inactive mutant of Nrdc in Nrdc−/−^EA^ iMEF. The EA mutant protein only retained H3K9ac levels in the promoter of Src among the 10 Nrdc target genes tested, in spite of the similar expression levels of the wild-type and mutant proteins in Nrdc−/−^WT^ and Nrdc−/−^EA^ iMEF, respectively (Fig. S4A). These results indicate that the enzymatic activity of Nrdc modulates histone acetylation, although the transcription of cell cycle-related Nrdc target genes, except for Ajuba, was rescued by the EA mutant protein. Taken together, these results indicate that Nrdc directly regulates the expression of cell cycle-associated genes in iMEF via the modulation of histone acetylation.

### Nrdc is involved in cell proliferation and cell cycle progression

**Figure 4:**
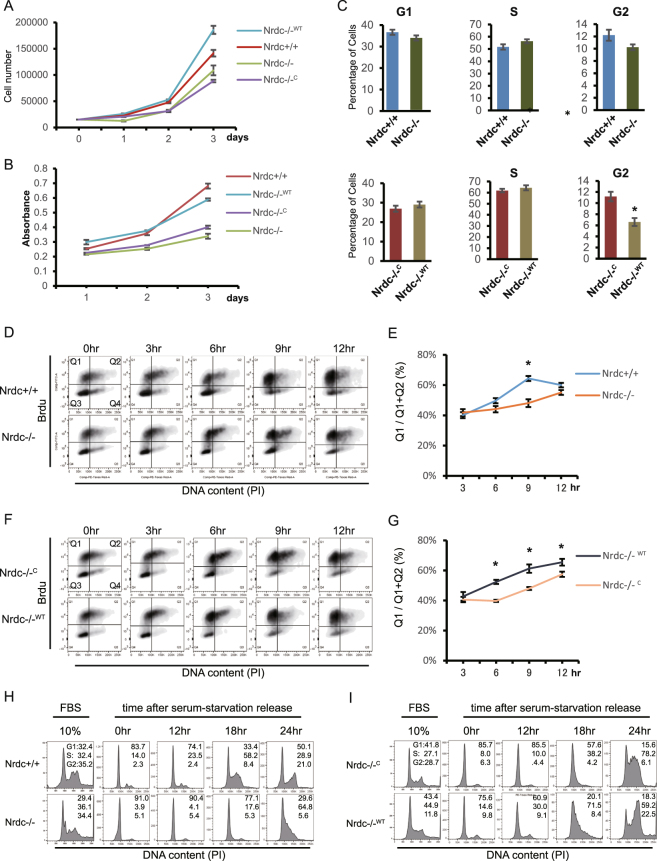
Nrdc is necessary for properly maintaining cell proliferation and cell cycle progression in iMEF. (**A**) Effects of Nrdc on cell proliferation. Each type of iMEF was plated on a 35-mm dish, and cell numbers were counted on days 1, 2, and 3. (**B**) The WST-8 cell growth assay was performed for each type of iMEF as described in (**A**). (**C**) Proportion of cells in each cell cycle phase estimated by the BrdU/PI FACS profiles (0 hr in Fig. 4D) of the Nrdc+/+ v.s. Nrdc−/− group (Upper) and Nrdc−/−^C^ v.s. Nrdc−/−^WT^ (Lower) group. G1 indicates the percentage of the population in Q4, S indicates Q1 + Q2, and G2 indicates Q3, respectively. (**D**–**G**) Each type of iMEF was labeled with BrdU and then analyzed by FACS at the indicated time point. Similar results were obtained in 3 independent experiments and representative images are shown (**D**,**F**). The percentage of Q1/(Q1 + Q2) was calculated in order to estimate the speed of entering the next G1 phase (n = 3 per group) (**E**,**G**). (**H**,**I**) Analysis of the cell cycle in each type of iMEF by flow cytometry. Cells under exponential growth (10% FBS) or cells released from serum starvation (time after serum-starvation release) were examined. All error bars indicate S.E. *Indicates p < 0.05.

Our integrative genome-wide analysis revealed that Nrdc regulates genes involved in the cell cycle. In order to assess how the perturbed expression of Nrdc affects the cell cycle and proliferation in iMEF, we examined the proliferative status of Nrdc+/+, Nrdc−/−, Nrdc−/−^WT^, and control virus vector-infected Nrdc−/−^C^ iMEF. The proliferation of Nrdc−/− iMEF was markedly weaker than that of Nrdc+/+ iMEF (Fig. 4A). Furthermore, decreased proliferation was clearly restored by the reintroduction of Nrdc (Nrdc−/−^WT^ iMEF), suggesting that decreased proliferation was mediated by Nrdc (Fig. 4B). In order to identify which stage of the cell cycle was affected by Nrdc, we performed BrdU incorporation analyses on asynchronously growing cells. No significant differences were observed in the proportion of cells in each cell cycle between Nrdc+/+ and Nrdc−/− iMEF, suggesting that Nrdc regulates the lengths of multiple cell cycle stages. On the other hand, Nrdc−/−^WT^ showed a more specific decrease in the G2/M fraction than Nrdc−/−^C^ iMEF (Fig. 4C,D, and F).

In order to specifically assess the rate of cell cycle progression from the S to the next G1 phase, BrdU-labeled iMEF were chased every 3 hr after labeling until 12 hr. Cells labeled during the S phase passed through the G2/M phase (Q2) and transferred to the G1 phase (Q1). Nrdc+/+ iMEF showed a significantly higher proportion of BrdU-labeled/low PI cells (Q1/[Q1 + Q2]) at 9 hr, indicating that more cells completed the M phase before 9 hr after labeling in Nrdc+/+ than in Nrdc−/− iMEF (Fig. 4D,E). Similarly, Nrdc−/−^WT^ showed a higher proportion of Q1/[Q1 + Q2] at 6, 9 and 12 hr, suggesting a significantly faster rate of cell cycle progression, particularly in the G2/M phase, in Nrdc−/−^WT^ than in Nrdc−/−^C^ iMEF (Fig. 4F,G).

In order to investigate whether Nrdc expression also affects G1/S transition, cell lines were synchronized in the G1 phase by serum starvation, then re-stimulated with 10% serum containing medium. As shown in Fig. 4H, Nrdc−/− iMEF showed the slower shifting of the peak corresponding to the S-phase fraction between 18 and 24 hr after the stimulation. The ectopic reintroduction of Nrdc (Nrdc−/−^WT^ iMEF) reproducibly increased the rate of G1/S transition more than Nrdc−/−^C^ iMEF (Fig. 4I). These results indicate that an Nrdc deficiency disturbed the entry of quiescent cells into the G1/S phase. Taken together, our results suggest that Nrdc is involved in cell cycle progression at multiple stages.

## Discussion

In the present study, we performed the first genome-wide analysis of Nrdc consisting of a transcriptome and ChIP-seq assay. This non-biased approach emphasized the role of Nrdc in epigenetic modifications and cell cycle regulation.

The first main result of this study is that Nrdc predominantly localizes to active promoter regions, which are characterized by specific histone modifications such as high levels of H3K4me2, H3K4me3, and H3K9ac. Consistent with this result, many Nrdc-binding genes exhibited high expression levels. Most Nrdc-binding genes encode proteins that participate in fundamental biological processes including protein translation, RNA metabolism, and the cell cycle (Table S2). We previously reported that Nrdc directly interacted with H3K4me2 and, to a lesser extent, H3K4me3^15^. Our ChIP seq analysis revealed that more than 90% of Nrdc-binding regions overlapped with H3K4me2-binding regions. However, detailed analyses demonstrated that Nrdc peaked at the center of the core promoter regions, whereas H3K4me2 showed the pattern of a broad dual peak around TSS. These distinct patterns suggest that H3K4me2 binding is not a sole means for Nrdc to recognize chromatin. We also previously showed that Nrdc associates with transcriptional modulator complexes, such as the SMRT/NCoR corepressor^15^ and PGC1-α coactivator^16^. Recent genome-wide studies revealed that these transcriptional complexes are distributed at active core promoter regions, which are similar to those of Nrdc^32,33^. Furthermore, binding sites for a number of transcriptional factors, such as ELK4 and JUNB (Fig. 1F), were enriched within Nrdc-binding regions. These results support our hypothesis that Nrdc functions as a transcriptional coregulator.

Several recent studies suggested crosstalk between histone acetylation and methylation^34^. For example, H3 acetylation appears to be promoted through Chd1 binding to H3K4me2 and Yng1 binding to H3K4me3^35,36^, whereas the deacetylation of H3 may be induced via the interaction between H3K4me3 and Ing2. We previously demonstrated that Nrdc physically binds to HDAC3, a member of the SMRT/NCoR corepressor complex. Moreover, several Nrdc-binding sites were associated with decreased histone acetylation and the recruitment of HDAC3^15^. In the present study, however, our ChIP-seq experiments showed that global H3K9 acetylation levels at Nrdc-binding promoters were higher in Nrdc+/+ than in Nrdc−/− iMEF. On the other hand, global H3K4me2 levels at Nrdc-binding sites were reduced in the presence of Nrdc. More detailed analysis of H3K9ac and H3K4me2 signals among the promoters demonstrated the higher enrichment of H3K9ac in up-regulated Nrdc target genes and lower enrichment of H3K4me2 in down-regulated targets in the presence of Nrdc (Fig. S5B,C). These data suggested that Nrdc may activate or repress gene transcriptions through distinct mechanisms. Since one of the mechanisms facilitating histone acetylation is mediated by H3K4 methylation^37^, another possible explanation could be that Nrdc is required for the proper recruitment of proteins regulating crosstalk between histone acetylation and methylation.

By combining the data sets of ChIP-seq and gene expression, we identified 448 genes as candidates for the direct target genes of Nrdc. Among them, we found an extensive connection between Nrdc and genes integral to cell cycle regulation by a gene ontology analysis. We showed that most of the cell cycle-associated targets of Nrdc were transcriptionally activated. The promoters of these target genes consistently showed an increase in H3K9 acetylation. The mRNA and H3K9ac levels of these genes were significantly decreased in Nrdc−/− cells, but were rescued by the transfection of wild-type Nrdc. These results suggest direct roles for Nrdc in transcriptional regulation. Decreased H3K9ac levels in Nrdc−/− cells were not fully restored by the enzymatically-inactive mutant of Nrdc. However, the loss of peptidase activity did not affect the gene expression levels of these targets. This result implies that some of the transcriptional activity of Nrdc is independent of its effects on histone acetylation. Although the precise mechanism remains to be elucidated, we previously demonstrated the role of Nrdc enzymatic activity in transcriptional regulation^16,17^. The transcriptional activity of PGC-1α in iMEF, measured by the luciferase reporter assay using Gal4-PGC-1α, was repressed by Nrdc via its peptidase activity^16^. The Nrdc-induced up-regulation of Mafa mRNA in the rat β-cell line was not recapitulated by the enzymatic inactive mutant of Nrdc^17^. Due to the possible role of peptidase activity in histone acetylation, the further characterization of functional interactions between Nrdc and the enzymes involved in histone acetylation is needed. Future studies using inactive mutant knock-in mice will provide more detailed insights into the biological impact of the functions of Nrdc peptidase.

Knockout-rescue analyses in iMEF confirmed the important roles of Nrdc in the maintenance of cell proliferation and cell cycle progression. Our results clearly revealed that Nrdc is involved in multiple stages of cell cycle progression. This characteristic of Nrdc may be attributed to its multifaceted target genes such as Cdk1, Fbxo5, and Ccnf. Cdk1 interacts with cyclin B to drive the G2-M transition, while it binds to other cyclins to regulate G1 progression and G1-S transition^38^. Fbxo5 is required for cyclin B accumulation and mitotic entry^39^. Ccnf is involved in mitotic progression by regulating centrosomal function via the ubiquitination of CP110^40^. Nrdc may integrate the functions of these cell cycle-associated target genes by maintaining a proper epigenetic status at promoter regions. Previous studies from other groups also showed that the nuclear localization of Nrdc in oocytes was regulated in a cell cycle-dependent manner^41^. On the other hand, Nrdc in the extracellular space may be involved in the cell cycle via the activation of growth factors and cytokines. In gastric cancer cells, Nrdc has been shown to enhance the ectodomain shedding of TNF-α and subsequent activation of cell cycle-promoting genes, such as Ccnd1 and bcl2^18^. The mechanisms by which cells utilize nuclear and extracellular Nrdc for cell cycle regulation currently remain unclear because it is not possible to force the expression of Nrdc in only the nucleus or extracellular space. Future studies need to focus on discriminating between the direct and indirect effects of Nrdc on the transcription of cell cycle-associated genes. Taken together, our results emphasize the importance of Nrdc for maintaining a proper epigenetic status and cell growth.

## Methods

### Cell culture and establishment of iMEF lines

The generation of immortalized Nrdc+/+ and Nrdc−/− iMEF was previously described^13^. Nrdc−/− iMEF ectopically expressing the wild-type (WT) or enzymatically inactive mutant of Nrdc (EA) were established by the infection of a lentivirus vector for the expression of the wild-type (Nrdc−/−^WT^) or mutant of Nrdc (Nrdc−/−^EA^). A cDNA encoding an enzymatically inactive mutant of mouse Nrdc was obtained by substituting the Glu^235^ codon (GAG) with an Ala codon (GCG) using the PCR technique, as previously described^16,30^. As control iMEF (Nrdc−/−^C^), Nrdc−/− iMEF were infected with an empty lentivirus vector. The biological replicates of cells at 1-3 passages after viral infection were used in all experiments. Cells were cultured in DMEM supplemented with 10% fetal bovine serum (FBS) and antibiotics.

### Antibodies

A mouse anti-mouse Nrdc monoclonal antibody (clone #2E6) was generated by the immunization of Nrdc-deficient mice with recombinant mouse Nrdc in our laboratory. The protein antigen used for immunization corresponded to the C-terminal fragment (G^348^ to K^1163^) of mouse Nrdc, synthesized using a silkworm protein expression system (Sysmex). Clone #2E6 was the most efficient for chromatin immunoprecipitation among the different clones of the anti-Nrdc monoclonal antibody. Other antibodies were from the following sources: H3K4me2 (07–030, Millipore), H3K9ac (06–942 Millipore), and control IgG (2729S, CST).

### ChIP

ChIP was performed using the ChIP-IT Express kit (Active motif) according to the manufacturer’s protocol, with the following specifications to improve efficiency. Ten million cells per sample were collected for the ChIP experiment. Cross-linked nuclear pellets were subjected to sonication for 10 min using Covaris S2 (Covaris). Chromatin fragments were incubated with a specific antibody and protein G magnetic beads (Active motif) at 4 °C overnight with rotation. Antibodies against Nrdc, H3K4me2, H3K9ac, and control IgG were used. The beads were then washed with ChIP buffer1 and ChIP buffer2 (Active motif), followed by successive washes in high salt buffer (20 mM Tris-HCl, pH8.0, 500 mM NaCl, 2 mM EDTA, 0.1% SDS, and 1% Triton X-100). The beads were resuspended in 100 μl of elution buffer (10 mM Tris-HCl pH8.0, 1 mM EDTA, and 0.5% SDS) and eluted at 65 °C for 30 min with rotation. The eluents were then incubated with 10 ng/μl of RnaseA at 37 °C for 30 min, followed by reverse-cross-linking with 0.4 mg/ml of proteinase K at 65 °C for 6 hr with rotation. DNA was purified using the Min Elute PCR Purification Kit (QIAGEN).

### ChIP-sequencing

Purified DNA was quantified using the Agilent 2100 Bioanalyzer with a DNA high sensitivity kit (Agilent Technologies), and the same amount of DNA input was subjected to library preparation. Fragmented libraries were created using the NEBNext Fast DNA Library Prep Set for the Ion Torrent kit (New England Biolabs) according to manufacturer’s protocol with slight modifications. Briefly, ChIP DNA was end-repaired, adapter-ligated, and size-selected with Agencourt AMPure XP (Beckman Coulter Inc.) to 150–350 bp in length. DNA was PCR amplified with the Ion barcode adapter (Thermo Fischer Inc.) for 12 cycles. After amplification, DNA was purified with AMpure XP and checked for size distributions and amounts using the Agilent 2100 Bioanalyzer. High-throughput sequencing was performed using Ion Proton semiconductor sequencers (Thermo Fischer Inc.) according to the manufacturer’s instructions. Base calling was performed using Torrent Suite (Thermo Fischer Inc.).

Reads were aligned with Bowtie2 to mouse genome build mm9. Regarding peak calling, we only used reads with a mapping quality score (MAPQ) > 5. Approximately 25, 25, 17, and 7 million uniquely mapped reads were obtained for Nrdc, DNA input, H3K4me2 and H3K9ac, respectively (Table S11). Peak detection was performed with a model-based analysis of ChIP-seq (MACS v1.4.2) with default settings (p < 1*10^−5^)^42^. Peak annotation was performed using the ChIPseeker package^43^ with mouse genome sequences (mm9). Regarding the association of peaks with specific promoters, peaks within −5 kb to+5 kb of TSS were defined as promoters. In TSS annotation, the longest transcript based on Ensembl exon locus annotation from BioMart was taken as the representative for each gene. The genes filtered against Ensembl protein-coding genes were used in further analyses. In the histone modification comparative analysis, the compEPItools package^44^ was used to normalize the numbers and lengths of the reads, and count the aligned reads in the promoter regions. ChIP-seq datasets for H3K4me3, H3K27me3, H3K36me3, CTCF, and DNA input were downloaded from a public database (GSE36048).

### RNA extraction and RT-PCR analysis

Total RNA was extracted using a combination of TRIzol® Reagent (Thermo Fischer Inc.) and the RNeasy mini kit (QIAGEN) according to the RNeasy protocol with TRIzol instead of QIAzol. First-strand cDNA was synthesized from total RNA using the Transcriptor First Strand cDNA Synthesis Kit (Roche). RT-PCR was performed using the LightCycler 96 system (Roche) and THUNDERBIRD qPCR (TOYOBO) following the manufacturer’s directions. The results obtained were standardized for comparisons by measuring the level of Gapdh mRNA in each sample. The primers used are listed in S1 Table.

### ChIP-PCR analysis

ChIP samples were analyzed by RT-PCR. The results obtained were normalized to DNA input. All primers used in this study are listed in S1 Table.

### cDNA library preparation and RNA-seq analysis

The Dynabeads® mRNA Direct kit (Thermo Fischer Inc.) was used to purify mRNA from 5 μg of total RNA according to the manufacturer’s instructions. We used the Ion Xpress Plus Fragment Library Kit (Thermo Fischer Inc.) to prepare a cDNA library according to the manufacturer’s instructions. Samples were sequenced using Ion Proton semiconductor sequencers (Thermo Fischer Inc.) according to the manufacturer’s instructions. We aligned raw reads to the mouse reference genome (Ensembl, mm9) using the spliced read aligner STAR^45^, then took any unmapped reads to Bowtie2 in the local mode^46^. Raw read counts were calculated by featureCounts^47^ in the Subread package. The detection of DEG and calculation of rpkm (reads per kilobase of exon per million mapped sequence reads) were performed using the edgeR package^48^ with Ensembl genes, filtered against protein-coding genes. In order to visualize gene expression, the rpkm of genes was log2 transformed with primary counts of 0.25, mean centered between Nrdc−/− and Nrdc−/−^WT^ iMEF, and plotted with the gplots package.

### Gene ontology analysis

DAVID Bioinformatics Resources (6.7) was used to analyze the Ensembl Gene IDs of Nrdc direct targets in order to detect enriched biological processes. Default settings were used for the GO analysis.

### Immunoblotting

Cells were washed twice with PBS, then harvested by scraping in lysis buffer (10 mM Tris-HCl, 1 mM EDTA, 130 mM NaCl, 1% NP-40, protease inhibitor cocktail, and phosphatase inhibitors [Sigma]). Scraped cells were lysed on ice for 10 min, and centrifuged for 10 min at 12000 rpm. The supernatants were then separated with LDS buffer (Thermo Fischer Inc.) and transferred to nitrocellulose filters. After blocking, filters were incubated with primary antibodies, followed by horseradish peroxidase-conjugated secondary antibodies. Immobilized peroxidase activity was detected with the enhanced chemiluminescence system (ECL, Amersham).

### Immunocytochemistry

Immunocytochemistry was performed as described previously^12^. Briefly, fixed cells were incubated with the rat monoclonal anti-mouse Nrdc antibody (#135), followed by an incubation with the secondary antibody (an Alexa Fluor 488-conjugated goat antibody to mouse IgG) and counterstaining with DAPI. Pictures of immunostained sections were acquired using the BZ-9000 digital microscope (Keyence).

### Cell proliferation assay

Each type of iMEF was plated on 35-mm culture dishes (1.5 × 10^4^/dish) and cell numbers in a dish were counted daily for 3 days using a Z1 coulter particle counter (Beckman Coulter) to extrapolate growth curves. Cell proliferation was also measured using Cell Counting Kit-8 (Dojindo Molecular Technologies) according to the manufacturer’s instructions. In the assay, cells were plated on 96-well dishes (5 × 10^3^/well) and cell proliferation was measured daily for 3 days following the manufacturer’s protocol.

### BrdU analysis

Asynchronously growing cells plated on 60-mm dishes were used for the BrdU (Bromodeoxyuridine) analysis. Cells were incubated with BrdU (50 μM) for 30 min and then collected by trypsinization 0, 3, 6, 9, and 12 hr after the completion of the BrdU treatment. Cells were centrifuged at 3000 rpm for 1 min, washed twice with PBS, and fixed with 70% ice-cold ethanol overnight. Fixed cells were denatured with 2 M HCl for 30 min, and treated with 0.1 M boric acid at pH 8.5 at room temperature for 5 min. After washing with blocking buffer (1% bovine serum albumin and 0.5% Tween20 in PBS), cells were incubated with 1:100 of the anti-BrdU antibody conjugated with Alexa Fluor 488 (B35130, Thermo Fischer) in blocking buffer at room temperature for 60 min. Cells were then washed with blocking buffer and incubated with PI-RNAse buffer (50 μg/ml PI and 10 μg/ml RNAse-A in PBS) at room temperature for 30 min. FACS Aria IIa (BD Biosciences) was used for the analysis.

### Cell synchronization by serum starvation

Cells were synchronized in the G1 phase by culturing in medium containing 0.1% FBS for 72 hr. The serum concentration was then increased to 10% to allow cells to reenter the cell cycle. Cells were collected 0, 12, 18, and 24 hr after the exchange to 10% serum-containing medium. In the FACS analysis, cells were washed and fixed with 70% ice-cold ethanol overnight. After washing with PBS, cells were stained with PI-RNAse buffer at room temperature for 30 min, and subjected to the FACS analysis. The Dean Jett Fox model of Flowjo software (v10.2, © FlowJo, LLC) was used to calculate the proportion of G1, S, and G2 cells in each sample.

### Statistical analysis

All data are presented as the mean ± standard error (S.E). The Student’s *t*-test was performed to compare two groups. * and † indicate P < 0.05. The relationship of the relative enrichment of read densities among promoter regions between each ChIP-seq experiment was assessed using Pearson’s correlation coefficient r. The Wilcoxon rank-sum test was used to calculate the significance of differences in enrichment.

### Accession Codes

Data have been deposited in GEO with the following accession numbers: ChIP-seq, GSE90107; mRNA-seq, GSE90108.

## Electronic supplementary material

Dataset 1

Dataset 2

## References

[1] Wu H, Sun YE (2006). Epigenetic regulation of stem cell differentiation. Pediatric research.

[2] Kubicek, S. *et al*. The role of histone modifications in epigenetic transitions during normal and perturbed development. *Ernst Schering Research Foundation workshop*, 1–27 (2006). 10.1007/3-540-37633-x_1 16568946

[3] Shilatifard A (2008). Molecular implementation and physiological roles for histone H3 lysine 4 (H3K4) methylation. Current opinion in cell biology.

[4] Eissenberg JC, Shilatifard A (2010). Histone H3 lysine 4 (H3K4) methylation in development and differentiation. Developmental biology.

[5] Barski A (2007). High-resolution profiling of histone methylations in the human genome. Cell.

[6] Bannister AJ, Kouzarides T (2011). Regulation of chromatin by histone modifications. Cell research.

[7] Pelling AL, Thorne AW, Crane-Robinson C (2000). A human genomic library enriched in transcriptionally active sequences (aDNA library). Genome research.

[8] Chesneau V (1994). Isolation and characterization of a dibasic selective metalloendopeptidase from rat testes that cleaves at the amino terminus of arginine residues. The Journal of biological chemistry.

[9] Nishi E, Prat A, Hospital V, Elenius K, Klagsbrun M (2001). N-arginine dibasic convertase is a specific receptor for heparin-binding EGF-like growth factor that mediates cell migration. The EMBO journal.

[10] Hiraoka Y (2007). Enhancement of alpha-secretase cleavage of amyloid precursor protein by a metalloendopeptidase nardilysin. Journal of neurochemistry.

[11] Hiraoka Y (2008). Ectodomain shedding of TNF-alpha is enhanced by nardilysin via activation of ADAM proteases. Biochemical and biophysical research communications.

[12] Nishi E, Hiraoka Y, Yoshida K, Okawa K, Kita T (2006). Nardilysin enhances ectodomain shedding of heparin-binding epidermal growth factor-like growth factor through activation of tumor necrosis factor-alpha-converting enzyme. The Journal of biological chemistry.

[13] Ohno M (2009). Nardilysin regulates axonal maturation and myelination in the central and peripheral nervous system. Nature neuroscience.

[14] Ohno M (2014). Nardilysin prevents amyloid plaque formation by enhancing alpha-secretase activity in an Alzheimer’s disease mouse model. Neurobiology of aging.

[15] Li J (2012). Identification and characterization of nardilysin as a novel dimethyl H3K4-binding protein involved in transcriptional regulation. The Journal of biological chemistry.

[16] Hiraoka Y (2014). Critical roles of nardilysin in the maintenance of body temperature homoeostasis. Nature communications.

[17] Nishi K (2016). Nardilysin Is Required for Maintaining Pancreatic beta-Cell Function. Diabetes.

[18] Kanda K (2012). Nardilysin and ADAM proteases promote gastric cancer cell growth by activating intrinsic cytokine signalling via enhanced ectodomain shedding of TNF-alpha. EMBO Mol Med.

[19] Choong LY (2011). Elevated NRD1 metalloprotease expression plays a role in breast cancer growth and proliferation. Genes, chromosomes & cancer.

[20] Uraoka N (2014). NRD1, which encodes nardilysin protein, promotes esophageal cancer cell invasion through induction of MMP2 and MMP3 expression. Cancer science.

[21] Csuhai E, Chen G, Hersh LB (1998). Regulation of N-arginine dibasic convertase activity by amines: putative role of a novel acidic domain as an amine binding site. Biochemistry.

[22] Huang da W, Sherman BT, Lempicki RA (2009). Systematic and integrative analysis of large gene lists using DAVID bioinformatics resources. Nature protocols.

[23] Zullo JM (2012). DNA sequence-dependent compartmentalization and silencing of chromatin at the nuclear lamina. Cell.

[24] Kundaje A (2012). Ubiquitous heterogeneity and asymmetry of the chromatin environment at regulatory elements. Genome research.

[25] Machanick P, Bailey TL (2011). MEME-ChIP: motif analysis of large DNA datasets. Bioinformatics (Oxford, England).

[26] Bailey TL, Machanick P (2012). Inferring direct DNA binding from ChIP-seq. Nucleic acids research.

[27] Sharrocks AD (2001). The ETS-domain transcription factor family. Nature reviews. Molecular cell biology.

[28] Shaulian E, Karin M (2002). AP-1 as a regulator of cell life and death. Nature cell biology.

[29] Wu C, Macleod I, Su AI (2013). BioGPS and MyGene.info: organizing online, gene-centric information. Nucleic acids research.

[30] Hospital V (1997). Human and rat testis express two mRNA species encoding variants of NRD convertase, a metalloendopeptidase of the insulinase family. Biochem J.

[31] Sims RJ, Reinberg D (2006). Histone H3 Lys 4 methylation: caught in a bind?. Genes & development.

[32] Raghav SK (2012). Integrative genomics identifies the corepressor SMRT as a gatekeeper of adipogenesis through the transcription factors C/EBPbeta and KAISO. Molecular cell.

[33] Charos AE (2012). A highly integrated and complex PPARGC1A transcription factor binding network in HepG2 cells. Genome research.

[34] Taverna SD (2007). Long-distance combinatorial linkage between methylation and acetylation on histone H3 N termini. Proceedings of the National Academy of Sciences of the United States of America.

[35] Pray-Grant MG (2005). Chd1 chromodomain links histone H3 methylation with SAGA- and SLIK-dependent acetylation. Nature.

[36] Martin DG (2006). The Yng1p plant homeodomain finger is a methyl-histone binding module that recognizes lysine 4-methylated histone H3. Molecular and cellular biology.

[37] Zhang Y, Reinberg D (2001). Transcription regulation by histone methylation: interplay between different covalent modifications of the core histone tails. Genes & development.

[38] Hu X, Moscinski LC (2011). Cdc2: a monopotent or pluripotent CDK?. Cell proliferation.

[39] Reimann JD (2001). Emi1 is a mitotic regulator that interacts with Cdc20 and inhibits the anaphase promoting complex. Cell.

[40] Klein DK (2015). Cyclin F suppresses B-Myb activity to promote cell cycle checkpoint control. Nature communications.

[41] Ma Z, Wang X, Hockman S, Snow EC, Hersh LB (2005). Subcellular localization of nardilysin during mouse oocyte maturation. Archives of biochemistry and biophysics.

[42] Zhang Y (2008). Model-based analysis of ChIP-Seq (MACS). Genome biology.

[43] Yu G, Wang LG, He QY (2015). ChIPseeker: an R/Bioconductor package for ChIP peak annotation, comparison and visualization. Bioinformatics (Oxford, England).

[44] Kishore K (2015). methylPipe and compEpiTools: a suite of R packages for the integrative analysis of epigenomics data. BMC bioinformatics.

[45] Dobin A (2013). STAR: ultrafast universal RNA-seq aligner. Bioinformatics (Oxford, England).

[46] Langmead B, Salzberg SL (2012). Fast gapped-read alignment with Bowtie 2. Nature methods.

[47] Liao Y, Smyth GK, Shi W (2014). featureCounts: an efficient general purpose program for assigning sequence reads to genomic features. Bioinformatics (Oxford, England).

[48] Robinson MD, McCarthy DJ, Smyth G (2010). K. edgeR: a Bioconductor package for differential expression analysis of digital gene expression data. Bioinformatics (Oxford, England).

