# Supplementary material for: Genome-wide profiling of nardilysin target genes reveals its role in epigenetic regulation and cell cycle progression
Source: Sci Rep. 2017 Nov 1;7:14801. doi: 10.1038/s41598-017-14942-4 (PMC5665917; doi:10.1038/s41598-017-14942-4)
Supplement: Supplementary file 1 — Dataset 1 [file 41598_2017_14942_MOESM1_ESM.pdf]

Supplementary Information

# Genome-wide profiling of nardilysin target genes reveals its role in epigenetic regulation and cell cycle progression

Yusuke Morita[1], Mikiko Ohno[1, 2], Kiyoto Nishi[1], Yoshinori Hiraoka[3], Sayaka Saijo[1], Shintaro Matsuda[1], Toru Kita[4], Takeshi Kimura[1], and Eiichiro Nishi[2 *]

[1] Department of Cardiovascular Medicine, Kyoto University Graduate School of Medicine,
54 Shogoin-Kawahara-cho, Sakyo-ku, Kyoto, 606-8507 Japan
[2] Department of Pharmacology, Shiga University of Medical Science
Seta Tsukinowa-cho, Otsu, Shiga 520-2192, Japan
[3] Division of Clinical Pharmacy, Faculty of Pharmaceutical Sciences, Kobe Gakuin University, Chuo-ku, Kobe 650-8586, Japan.
[4] Kobe Home Medical and Nursing Care Promotion Foundation, 14-1 Naka Ichiriyama, Kami Aza, Shimotani, Yamada-cho, Kita-ku, Kobe, 651-1102, Japan
* enishi@belle.shiga-med.ac.jp, nishi@kuhp.kyoto-u.ac.jp

Fig.S1

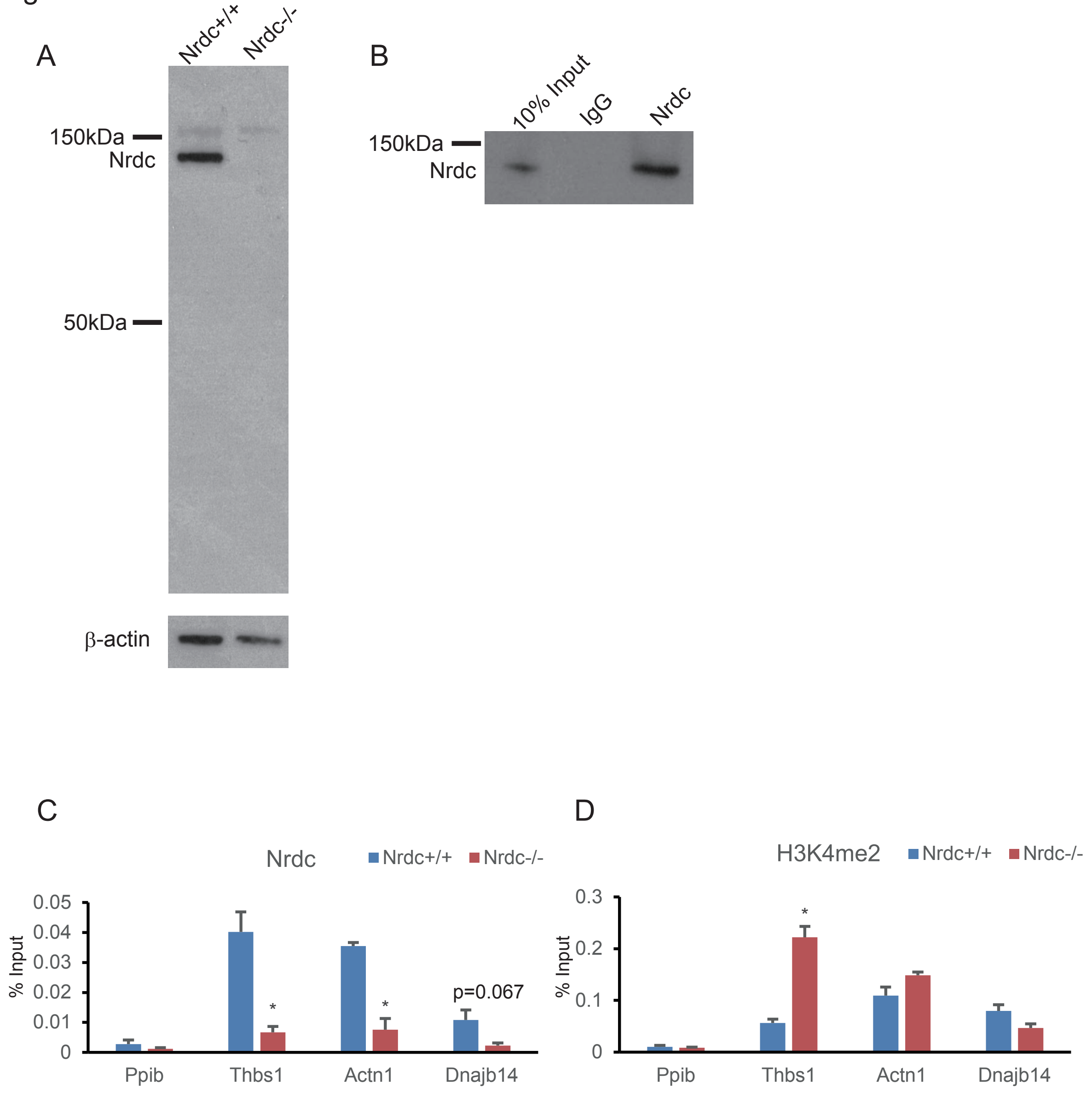


**Fig. S1. Validation of a mouse monoclonal anti-Nrdc antibody (#2E6) for ChIP-seq.**

(A) A Western blot analysis of cross-linked chromatin samples from Nrdc+/+ and Nrdc-/- iMEF shows the high specificity of the anti-Nrdc antibody. (B) Samples obtained by immunoprecipitation with the anti-Nrdc antibody (#2E6) or control IgG of cross-linked chromatin samples from Nrdc+/+ iMEF were probed with the anti-Nrdc-antibody (#135).

(C, D) ChIP-PCR analysis using anti-Nrdc (C) and anti-H3K4me2 (D) antibody in Nrdc+/+ and Nrdc-/- iMEF (n=3 per group). Target genes (Thbs1, Actn1, Dnajb14) and negative control gene (Ppib) were randomly selected from H3K4me2-enriched and H3K4me2-negative genome regions, respectively.

* indicates $p < 0.05$ between Nrdc+/+ v.s. Nrdc-/- group.

Fig.S2

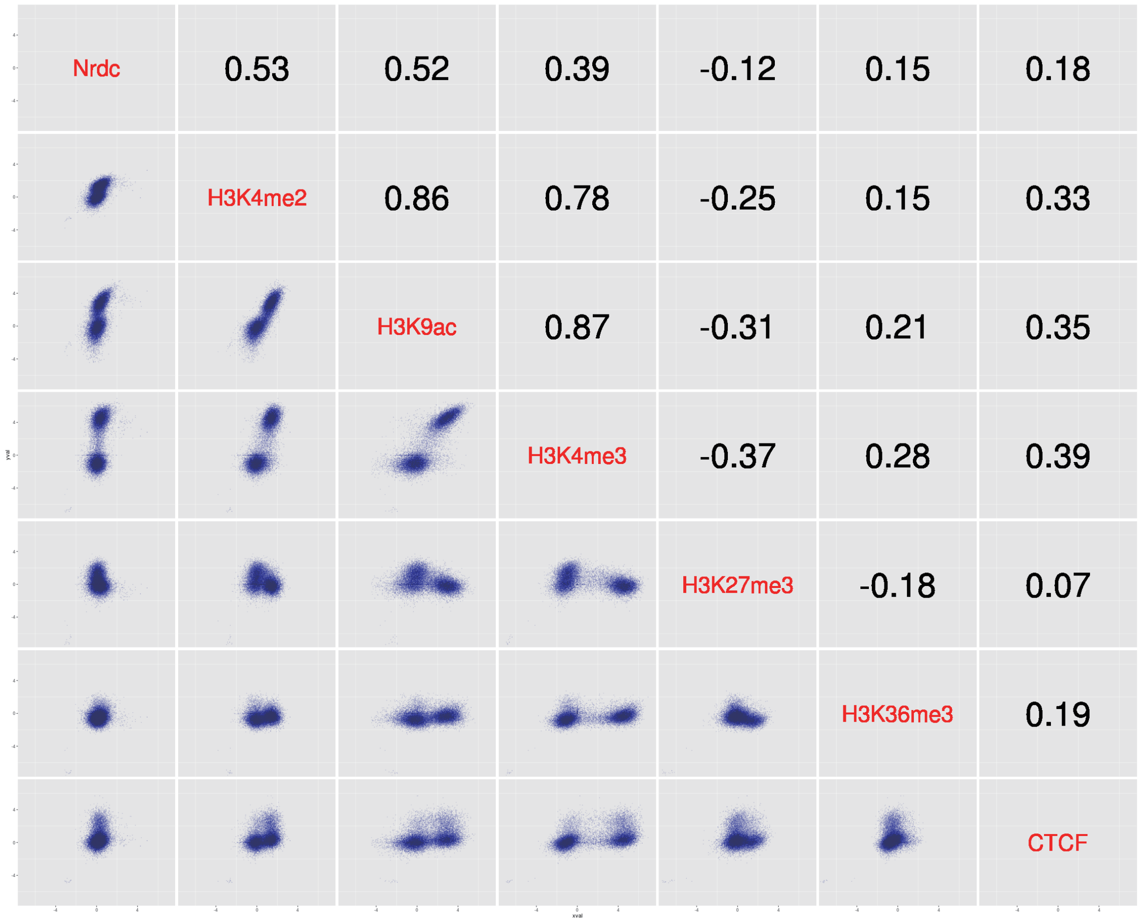


**Fig. S2. Correlation analysis of the enrichment of Nrdc and histone marks in iMEF.**

Correlation analysis of the relative enrichment of Nrdc and indicated histone marks (H3K4me2, H3K9ac, H3K4me3, H3K27me3, H3K36me3, and CTCF transcriptional factor) in promoters of Ensembl genes in Nrdc+/+ iMEF. Pearson's r values are shown.

Fig.S3

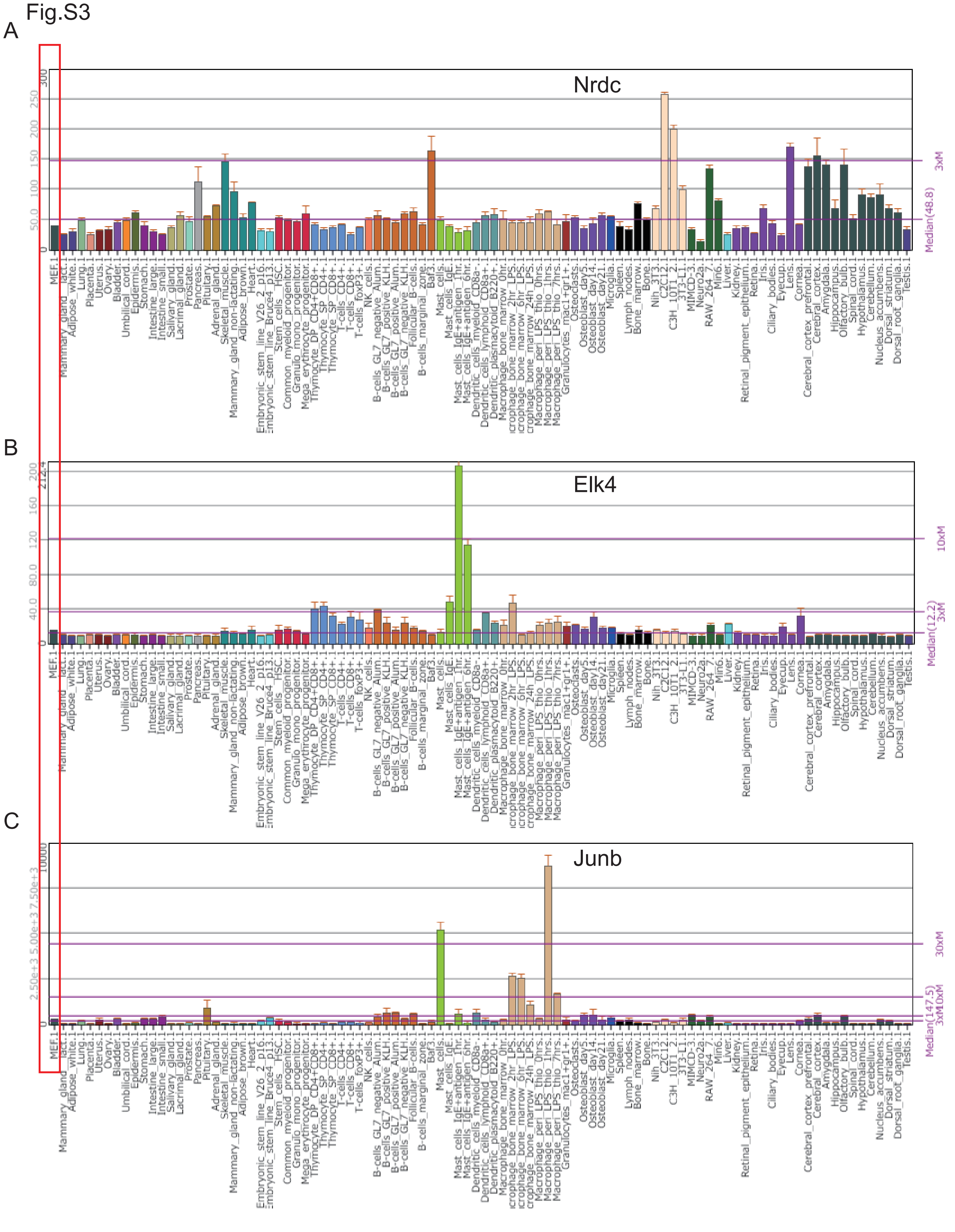


**Fig. S3. Expression profiles of Nrdc, Elk4, and Junb in mice tissues from BioGPS.** (A) Nrdc is ubiquitously expressed in mice tissues and cell lines. (B, C) Elk4 and Junb are specifically expressed in mast cells and macrophages, respectively.

Fig.S4

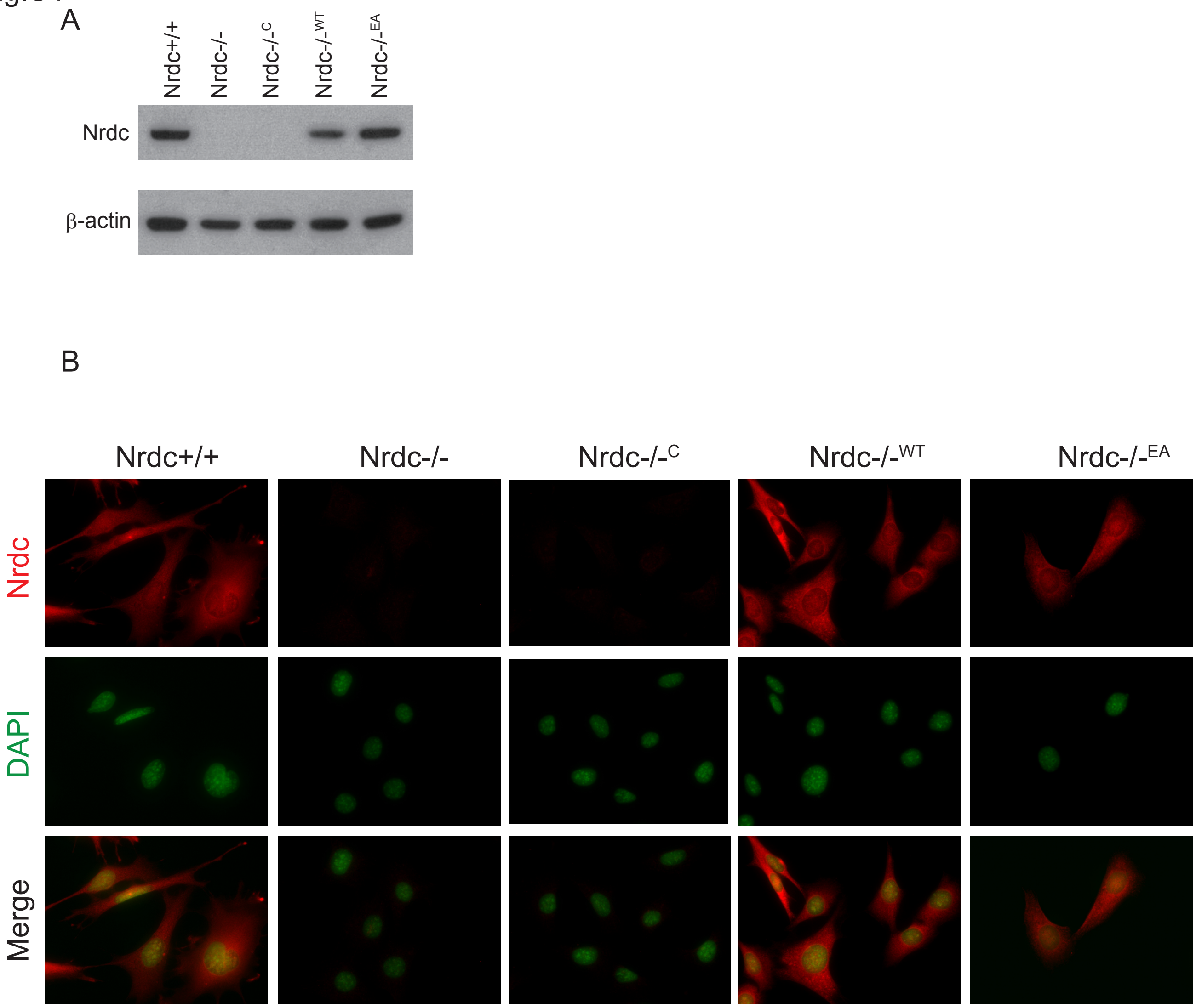


**Fig. S4. Validation of Nrdc protein expression in each iMEF.**

(A) Western blotting showing the protein levels of Nrdc and β-actin in Nrdc+/+, Nrdc-/-, Nrdc-/-$^{C}$, Nrdc-/-$^{WT}$, and Nrdc-/-$^{EA}$ iMEF. (B) Immunocytochemistry of Nrdc in each iMEF showing the similar localization pattern of Nrdc in Nrdc+/+, Nrdc-/-$^{WT}$, and Nrdc-/-$^{EA}$ iMEF. DAPI was used to visualize the nucleus.

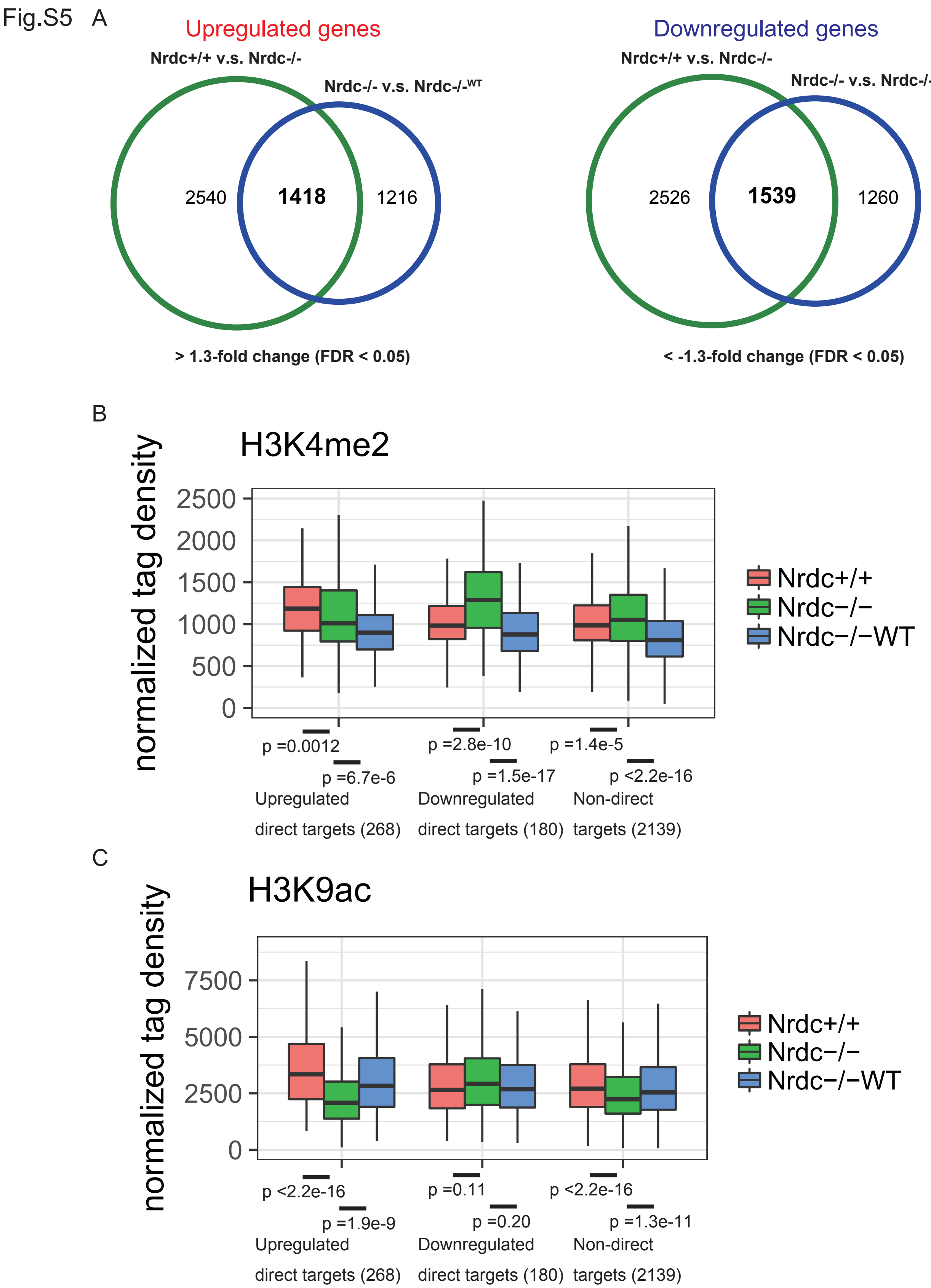


**Fig. S5. Separate Analyses of histone modifications in the promoter of genes up-regulated or down-regulated by Nrdc.**

(A) Separate Venn diagrams for genes up-regulated (left) or down-regulated (right) by Nrdc expression (Separate analysis of Venn diagram shown in Figure 2C).

(B, C) Separate analysis of H3K4me2 (B) and H3K9ac (C) levels in the promoters of i) up-regulated direct targets (268), ii) down-regulated direct targets (180), and iii) non-direct targets (2139).

Fig.S6

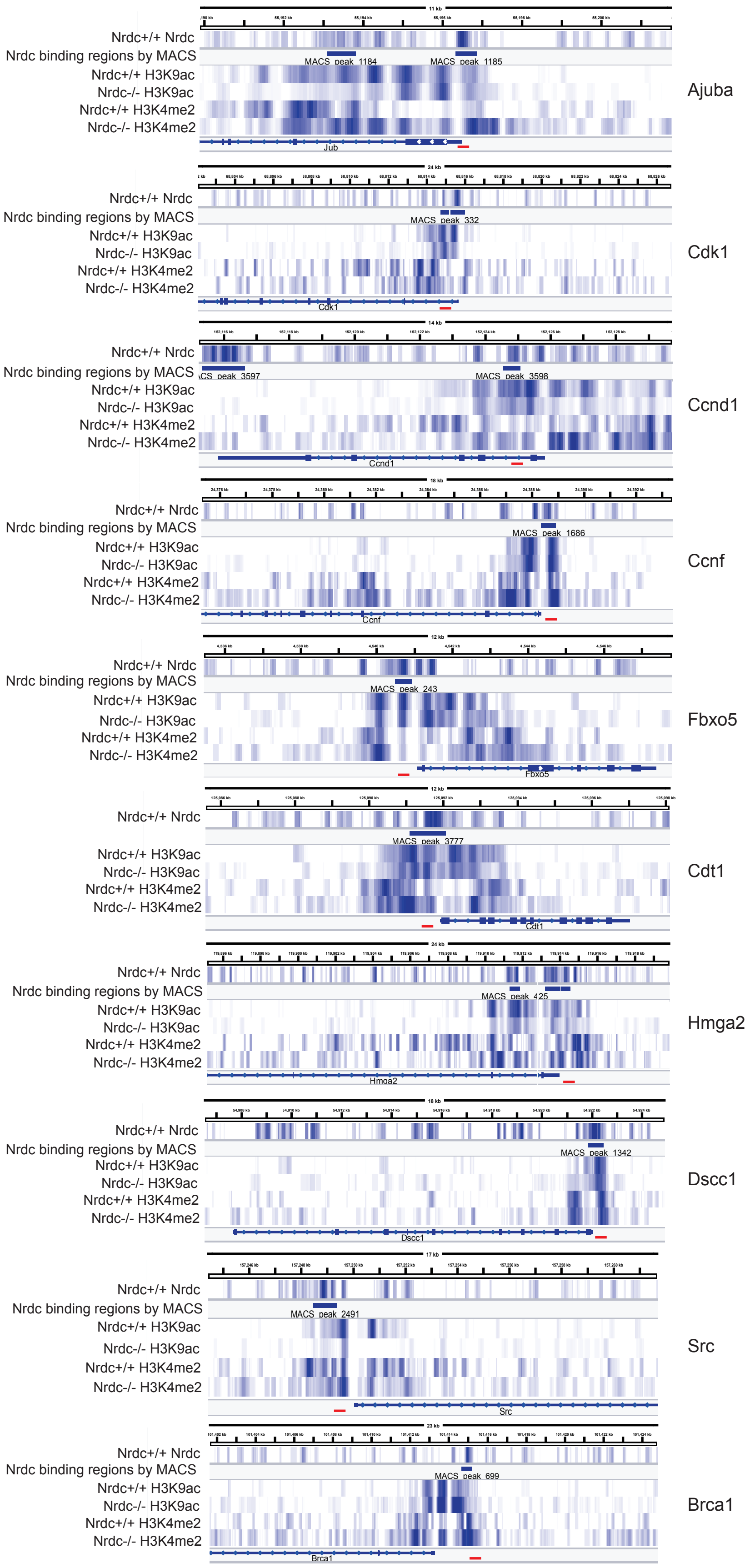


**Fig.S6. Heatmaps of Nrdc, H3K4me2, and H3K9ac binding regions.**

(A) Heatmaps showing the distribution of Nrdc, H3K4me2, and H3K9ac ChIP-seq signals around the Nrdc direct target promoters in Nrdc+/+ and Nrdc-/- iMEF. Nrdc binding regions detcted by MACS were also shown. Red bars indicate the specific regions for primers in ChIP-PCR experiments described in Fig. 4C-G. The color scale for the heatmap is identical between Nrdc+/+ and Nrdc-/- groups.

Fig.S7

Fig. S1A

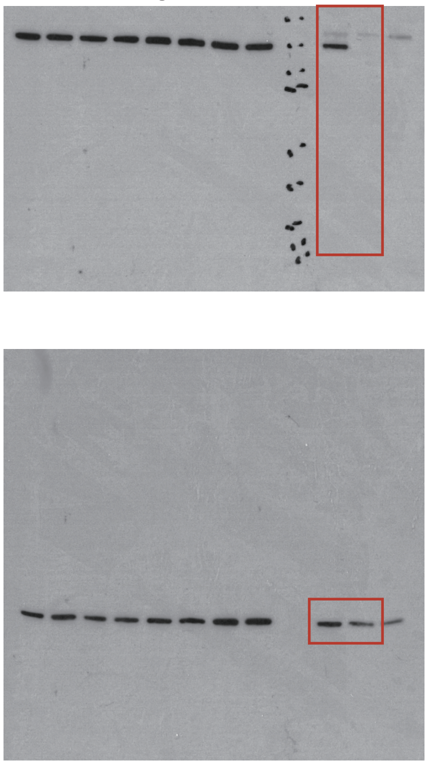

Fig. S1B

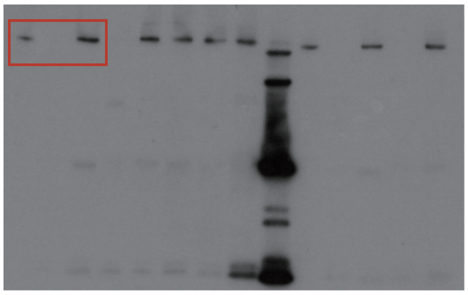

Fig. S4A

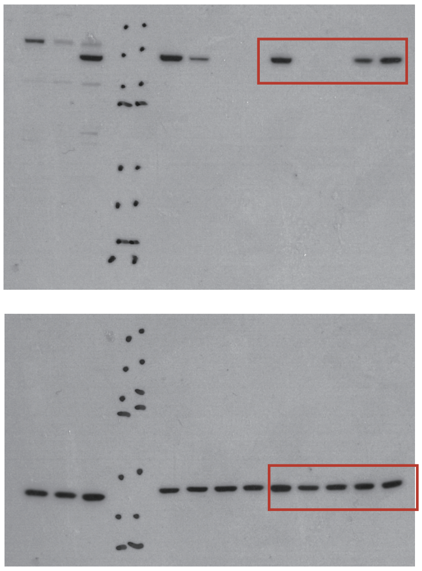

**Fig. S7. Uncropped gels and western blots.**

The original immunoblot data that were acquired by X-ray films are shown (Fig. S1 and S4).
